# A Virtual Reality Simulation of Drug Users’ Everyday Life: The Effect of Supported Sensorimotor Contingencies on Empathy

**DOI:** 10.3389/fpsyg.2020.01242

**Published:** 2020-06-05

**Authors:** Maria Christofi, Despina Michael-Grigoriou, Christos Kyrlitsias

**Affiliations:** ^1^GET Lab, Department of Multimedia and Graphic Arts, Cyprus University of Technology, Limassol, Cyprus; ^2^Research Centre on Interactive Media Smart Systems and Emerging Technologies – RISE, Nicosia, Cyprus

**Keywords:** virtual reality, sensorimotor contingencies, perspective-taking, empathy, attitudes

## Abstract

Perspective taking techniques have been used to transport people into imaginary situations and the lives of others. Virtual Reality provides an immersive way to virtually experience the lives of stigmatized by society members. Through the support of sensorimotor contingencies, people can use natural movements to view and interact with the virtual world around them. In this study, we compared a perspective-taking immersive Virtual Reality system which supports a number of sensorimotor contingencies (SC group) with a perspective-taking desktop system of the same application but without support of any sensorimotor contingencies (NSC group), to investigate the effect of the supported sensorimotor contingencies in promoting empathy and positive attitudes toward drug users. Results demonstrate a strong correlation between closeness to the drug user and empathy in the SC group. In both groups there were a within group significant change in their reported attitudes before and after their exposure. Finally, participants in the SC condition reported significantly higher levels of Place Illusion (PI), body ownership, agency and plausibility of people. Further research is needed to investigate how sensorimotor contingencies can be exploited to the fullest to be used as an effective method to induce empathy and change attitudes toward stigmatized by society people.

## Introduction

Members of stigmatized groups are often discriminated against in their workplace, educational settings, health care, and the criminal justice system (Sidanius and Pratto, 2001). A reliable method that has been shown in reducing negative social stereotyping is perspective taking, which can be defined as the cognitive capacity to perceive the world from another person’s viewpoint (Davis, 1983; Perdue and Gurtman, 1990; Shechtman and Tanus, 2006; Galinsky et al., 2008; Tomasello, 2009; Zaki and Ochsner, 2012). Furthermore, extensive research by Dan Batson has shown that perspective taking can lead to an increase of empathy, and this can lead to prosocial behaviors toward not only members of stigmatized groups, but toward stigmatized groups as a whole (Batson et al., 1997, 2003; Lamm et al., 2007). Empathy can be defined as the ability to connect emotionally with another individual and understand his point of view (Davis, 1983; Galinsky et al., 2008).

The field of Virtual Reality (VR) offers new ways to induce empathy into people. As Slater and Sanchez-Vives (2016) state, “the primary technological goal of VR is to realize perception through natural sensorimotor contingencies to the best extent possible.” People can turn their heads, like in real life, to look around while wearing a head-mounted display (HMD), they can bend over to look under a table, they can reach out their hands to grab objects. With VR, we can show experiences from any point of view, therefore we no longer must rely on people’s imagination. We can create experiences that genuinely show people how it is to take the place of somebody else and create a narrative that unfolds around them and therefore people can focus on the events that are happening around them and how they make them feel and go through a more genuine experience of what is like to be that person. In VR, we can also elicit presence, the illusion of being in the virtual world (Place Illusion, PI), and the extent to which the situation and events seemed to be really happening (Plausibility Illusion, Psi) (Slater, 2009).

Bertrand et al.’s (2018) study, highlighted immersive embodied virtual reality (EVR) strategies for empathy. Their strategies included the Body ownership illusion through multisensory and motor perspective taking, which if induced, could modulate bias, mimicry, similarity and emotion after the experience. Also, the agency illusion through embodiment combining voluntary and involuntary actions, which could result in self-attribution of avatar’s actions. Another strategy was place and plausibility illusion through sensorimotor stimuli and a highly credible environment, which could result for the users to behave and feel as if they are in the VR environment. Lastly, they mentioned the Proteus Effect (Yee and Bailenson, 2007; Yee et al., 2009) through avatars presenting empathy-related traits and appearances for the reinforcement of positive or negative stereotypes and the modulation of behavior after the experience. It has been shown that the Proteus effect is mediated by the level of embodiment felt by users in relation to their avatar (Ash, 2016) suggesting that EVR can enhance this effect. Bertrand et al.’s (2018) argue that empathy training methods using avatars designed to improve empathy would induce beneficial behavioral changes and improve positive perceptions. They suggested the embodiment of a digital avatar of an outgroup member that presents traits that contradict stereotypes, which we have done in our study.

The use of immersive virtual environments to have an individual take on the perspective of another individual is known as Virtual Reality Perspective-Taking (VRPT) (van Loon et al., 2018). Ahn et al. (2013) conducted three experiments that explored whether embodied experiences via Immersive Virtual Environment Technology (IVET) would elicit greater self-other merging, favorable attitudes, and helping toward persons with disabilities compared to traditional perspective taking. Penn et al. (2010) conducted a 4-condition, between subjects’ experiment (*N* = 112), wherein participants were exposed to either a virtual simulation of schizophrenia, a written empathy-set induction of schizophrenia, a combination of both the simulation and written empathy conditions, or a control condition. In Schutte and Stilinović’s (2017), randomly assigned participants viewed a documentary featuring a young girl living in a refugee camp either in a virtual reality format or in a control two-dimensional format. Results indicated that the virtual reality experience resulted in greater engagement and a higher level of empathy for the refugee girl compared to the control condition. Chowdhury et al. (2019) study showed that a tracked HMD and a wheelchair interface had significantly larger effects on participants’ implicit association toward people with disabilities than a desktop monitor and a gamepad. Hamilton-Giachritsis et al. (2018) study investigated whether embodiment of mothers into the body of a young child and engaging in an interaction with a virtual mother facilitates perspective-taking and empathy. Results showed that experiencing negative maternal behavior increased levels of empathy and participants reported a strong body ownership illusion for the child body that led to cognitive, emotional and physical reactions.

Herrera et al. (2018) conducted two experiments to compare the short and long-term effects of a traditional perspective-taking task and a VR perspective-taking task and to explore the role of technological immersion when it comes to different types of mediated perspective-taking tasks, relating to the homeless. Seinfeld et al. (2018) used virtual reality to allow offenders to virtually embody a victim of domestic abuse. It has been showed by several studies (Groom et al., 2009; Farmer et al., 2012; Maister et al., 2013; Peck et al., 2013; Banakou et al., 2016; Behm-Morawitz et al., 2016; Hasler et al., 2017) that embodiment of light-skinned participants in a dark-skinned virtual body significantly reduced implicit racial bias against dark-skinned people. Finally, a study (Christofi et al., 2018; Stavroulia et al., 2018, 2019) examined the effect of viewing in VR, a drug incident at a school environment from different perspectives and viewpoints in relation to the level of presence achieved and the effects on mood states and empathy of teachers who were immersed in a virtual school where a drug use incident took place.

A survey regarding VR interventions for stigmatized groups has been made by Christofi and Michael-Grigoriou (2017), with additional emphasis on the measurements used for prejudice and empathy. The studies reviewed were grouped according to the social stigma form that the group investigated belonged to, as defined by Goffman (2009), which are; (i) overt or external deformations, (ii) deviation in personal traits, and (iii) tribal stigmas. The survey reviewed the measurements used in these studies for empathy and prejudice. It concluded that the majority of the studies focused on the third form of social stigma, and most specifically on reducing implicit racial bias against dark-skinned people. As far as the measurements used for empathy and prejudice, it was observed that in the reviewed studies they tended to use self-report instruments more and rarely used behavioral observational and neuroscientific methods which could be more accurate than self-reports from the participants.

Drug users were chosen as the social target in this study because they are considered an extreme outgroup that people often struggle to empathize with (Harris and Fiske, 2006), and it is a stigmatized group that hasn’t been studied much in this context before. In this study, we compared a VRPT system that supports a number of sensorimotor contingencies (SC condition) and a less immersive perspective-taking system using a desktop computer with a minimum support of sensorimotor contingencies (NSC condition), to investigate their effect in promoting empathy and creating positive attitudes toward drug users.

Following the strategies and suggestions of Bertrand et al.’s (2018), our hypothesis was that participants in the SC condition would be more empathetic after their experience, and have more positive attitudes toward drug users, and feel more emotionally close to them, than participants in the NSC condition.

## Materials and Methods

### Application

An application was designed and developed in which participants were able to virtually experience some scenes of the life of a person who became a drug user, through his point of view, in order to debunk stereotypes surrounding drug users. In total, the participants viewed nine scenes from the life of the drug user. The scenario and scenes they virtually experienced are described in more detail in the following sections.

A number of the scenes, have a mirror on one of the walls (including the first one for familiarization with the VR equipment and the suit), so that the participants could see the avatar they were embodying and progressively see some of the physical changes, the drug consumed by their avatar which in this scenario is cocaine, does to people, which include dilated pupils, increased heart rate, extreme weight loss, bloody nose and cocaine powder running through the nose. These changes were visible to their avatar from the scene after he consumed cocaine in one of the starting scenes (Scene 5 as described below).

The scenario put the participants in the shoes of a man named Mark, a 28-year old man, who recently lost his mother due to cancer, and is living with his wife Amanda. It is said in the narrative, that he is stressed due to his workplace, where his boss assigns him many duties. One day at work, Mark’s colleague sitting at the desk next to him, invited him to a party, to which he attends. A man there, offers Mark to try out a drug, in this instance; cocaine, for the first time, to “loosen up and have fun,” as the drug dealer tells him. Mark, due to dealing with his mother’s death and extensive work stress, obeys, follows that man to the bathroom, does drug use for the first time and becomes addicted to cocaine. Then, participants experience the consequences of this action which include his inability to work, which is shown as a scene at work where his boss yells at him for not doing his job right, a visit to his drug dealer and his changing relationship with his wife, who threatens him in the end of the narrative to leave him if he doesn’t seek help for his drug problem. A video illustrating the application can be found in the Supplementary Video S1.

#### Scenes

**Scene 1 – Bathroom:** You are in the bathroom, standing in front of the sink. There is a mirror in front of you, where you can look at your avatar. Amanda is standing close to you and informs him that she invited your friends for dinner, so that it takes your mind away from work and your mother’s loss.

**Scene 2 – Dinner**: You are in the dinner, and your friends are discussing sports on the table. Then one of your friends, Lisa, mentions that you are quiet tonight, followed by Amanda explaining that it is because of the work stress, among other stuff. In this scene, there is a mirror on the left side of the room, where participants can view their avatar.

**Scene 3 – Work #1**: You are in your office, and your boss is telling you to prepare some files for him before lunch time because important clients are waiting for them. Your boss leaves and your coworker turns to you and invites you to a party in a few days to release all your stress.

**Scene 4 – Party #1**: You are in that party, loud music is playing, people dancing, drinking around you. Your coworker is telling you to enjoy yourself, and then a man notices how stressed and serious your character is and tells you to follow him.

**FIGURE 1:**
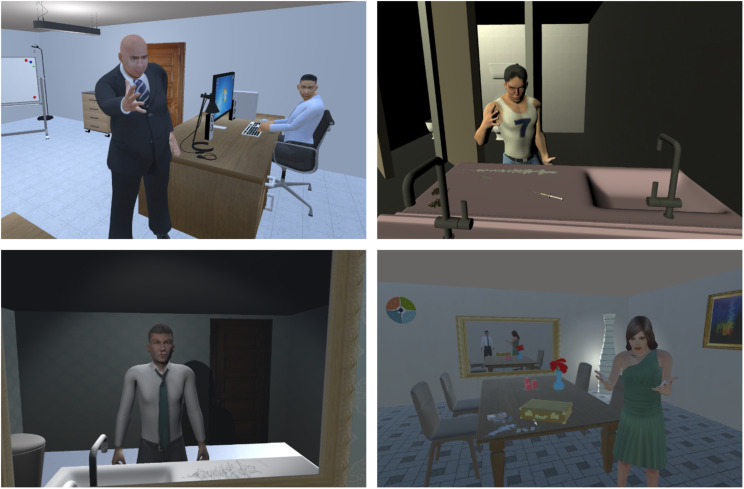
Workplace scene (top-left), party scene (top-right), bathroom scene, illustrating the physical effects of cocaine use (bottom-left), consequences on the relationship (bottom right).

**Scene 5 – Party #2**: You are in the toilets of the house that the party takes place in. In front of you there are two sinks with needles, cocaine powder divided in straight lines and razors. The man stands in front of the second sink, and sniffs cocaine and urges you to do the same (Figure 1, top right).

**Scene 6 – Drug dealer:** You stand in front of your drug dealer who is sitting relaxed at a table where you can see cocaine in lines, razors and a bag and money laying around in packs. The dealer wonders how you can afford all this cocaine, implying that you are a regular and warns you that demand is getting bigger and the prices are getting higher and next time you should bring more money.

Starting from this scene, participants can view the physical changes cocaine had done to their character, which include weight loss (in the starting scenes Mark appears to have a belly so that the change is more noticeable), so in this scene he looks really thin, his eyes are red, he has blood running from his nose, and cocaine power visible on the nose as well.

**Scene 7 – Work #2**: Your boss yells at you for not showing up to an important meeting the day before (Figure 1, top left) and then your coworker tells you to look at yourself in a mirror and that he worries about you.

**Scene 8 – Bathroom #2**: You locked yourself in the bathroom of your house (the same one you see in the first scene), and standing in front of the mirror, on the sink you can see cocaine powder and a razor (Figure 1, bottom left). You can listen to your wife, who is outside the room, wondering why you locked the door and informing you that the food is ready.

**Scene 9 – Dining area:** You stand in the dining area, your wife is standing by the table and you see your bag opened and cocaine on the table as well as money found by your wife, who is disappointed in you and says she noticed a big withdrawal of money from your shared account and it explains all the symptoms you have been showing lately (Figure 1, bottom right). She wonders what your mother would say about all this and urges you to seek some help or she is going to leave you.

The goal of showing the participants the whole journey of this man was to reverse the fundamental attribution error, that people tend to do. According to Ross (1977), the fundamental attribution error is defined as “the tendency for attributors to underestimate the impact of situational factors and to overestimate the role of dispositional factors in controlling behavior.” People tend to overestimate the influence of personality and underestimating the influence of situations, when explaining other people’s behavior (Myers, 2010). In this case and with our narrative, we wanted to show the participants, that in most cases situations drive people to drug use. According to the self-medication hypothesis (Khantzian, 1997, 2003), addictive drugs have appeal because during the short term they relieve painful feelings and psychological distress. There is a tendency in society to stigmatize drug users, as people think they use drugs for their enjoyment only. The character in the scenario is seen using drugs as a coping mechanism for the death of his mother and the stress from his work. Additionally, we followed the suggestion of Bertrand et al.’s (2018) for the embodiment of a digital avatar of an outgroup member that presents traits that contradict stereotypes, which we have done in our study. Additional screenshots from the scenes can be found in the Supplementary Table S1.

### Participants

A total number of 40 participants (*n* = 40) participated in the study, 21 of them male and 19 female. For the recruitment of the participants we used convenience sampling. Ages of participants varied from 18 to 59 with the majority (42.5%) in the range of 18–24 years old. Furthermore, data on participants’ experience in using VR environments, indicated that most of the participants (42.5%) had little experience with VR. A table with the frequencies of the demographics across the two groups can be found in Supplementary Table S2.

### Experimental Design

A between-groups design was used to conduct the study. The participants were randomly assigned into two groups, the Sensorimotor Contingencies condition (SC) (*n* = 20) and No Sensorimotor Contingencies (NSC) condition (*n* = 20), which are explained further in the following sections. The majority (11) of the participants in the NSC group were aged between (18–24), and for the SC group the majority (8) aged between 25 and 29 years old. Regarding occupation, in the NSC group the majority (12) were students and in the SC group (7) were working in the private sector. The study has been approved by the Research’s Ethics and Deontology committee of the Cyprus University of Technology. All participants provided their written informed consent to participate in this study. Written informed consent was obtained from the individuals for the publication of any potentially identifiable images or data included in this article.

#### SC Condition

This condition supports several sensorimotor contingencies for the user. It required the participants to put on a VR HMD allowing stereo viewing in 360 degrees of the surrounding environment and supporting head tracking allowing displacement of the viewing scene in a physical way. Real time tracking of full-body’s and hands’, at the detail of the fingers’, movements was also supported and real time mapping of the participant’s movements to the virtual avatar as well. That was achieved by having the participants fitted with a wireless full body motion capture suit and VR data gloves. Wearing both, the participants were virtually embodying the drug user. They were viewing the virtual world in first-person view and could rotate their head to change their point of view. There was no other stimulation than the first-person perspective view over the body.

The participants could not interact with the application in any way or with the other avatars or virtual items in the virtual world. The participants were required to be standing up and could move their body and head around to change their point of view and watch their avatar in the virtual world, which was the drug user, move simultaneously to their real time movements.

#### NSC Condition

The application of the NSC version was identical with the SC version with the difference being that the participants did not wear any of the equipment mentioned above thus no sensorimotor contingencies were supported. Instead, they were sitting on a chair in front of a desktop computer, viewing the same application, this time, through a flat computer screen and could only turn their point of view with the use of the mouse.

### Materials and Technical Setup

The application used for both groups was developed in the Unity software. The models of the avatars in the application were created using the online software Autodesk^®^ Character generator, in which a skeleton was also added to the models as well as facial blend shapes, so that their mouths could move according to the dialogues recorded which were recorded using a Philips audio recorder. These recordings were then edited in the Adobe Audition CS6 software.

**FIGURE 2:**
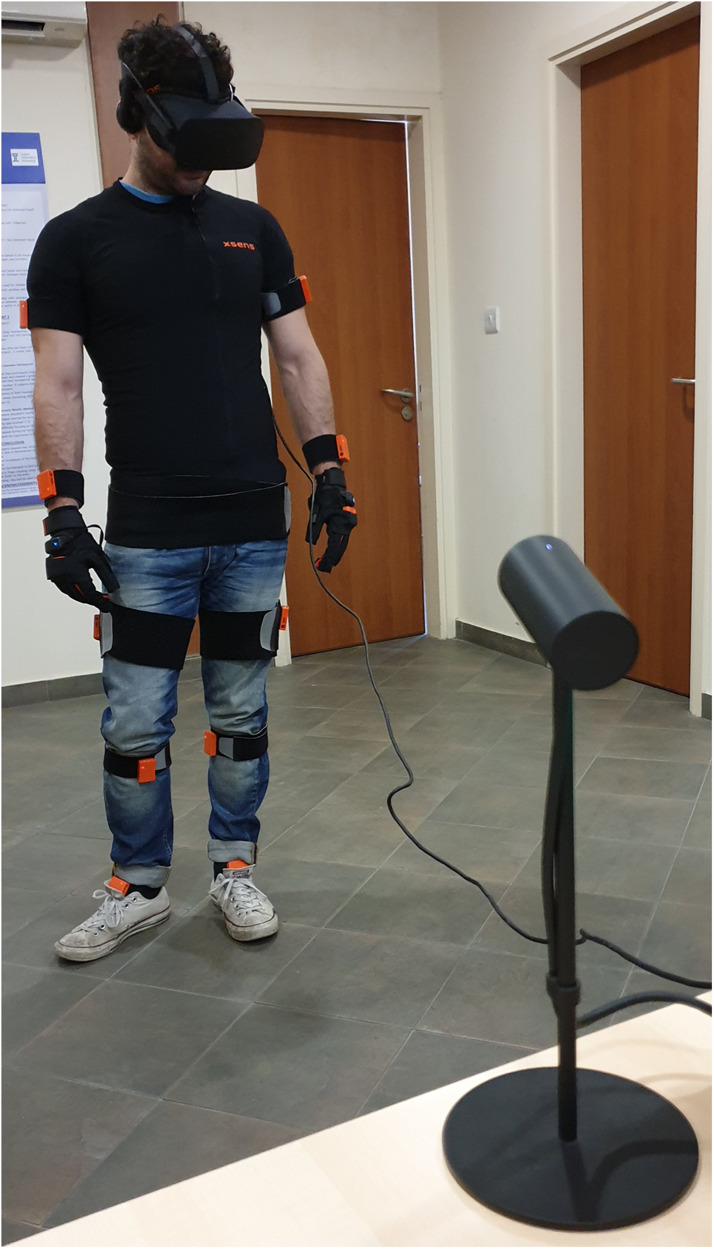
One of the participants of the SC condition wearing the Oculus Rift, Xsens Awinda trackers, and Manus VR gloves.

For the SC group the virtual environment was displayed through an Oculus Rift CV HMD. This has two 1080 × 1200 pixels OLEDs per eye at 90 Hz display refresh rate, coupled with a positional tracker and built-in headphones. The participants in this group had to wear the Xsens MVN Awinda wireless motion capture trackers for real time body movement tracking which was mapped to their avatar in the virtual world through the Xsens software and the Manus VR – Xsens Edition gloves which offered finger tracking (see Figure 2).

**FIGURE 3:**
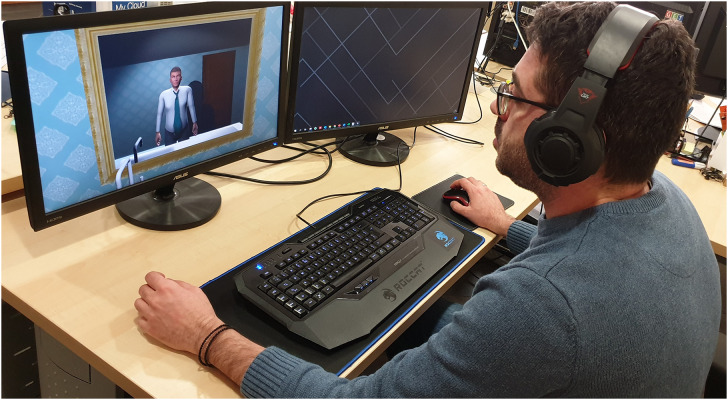
One of the participants of the NSC group.

For the NSC condition the application was displayed to participants through 1920 × 1080 pixels 24-inch computer screen. A computer mouse was used for the interaction with the application and headphones for the sound (see Figure 3).

### Procedure

Upon arriving at the laboratory, the participants were randomly assigned to one of the two groups, NSC or SC and were asked to read and sign the consent form. Then they were given the pre-questionnaire. Once this process was completed, participants in the SC group were asked to put on the VR HMD, Xsens Awinda trackers and VR gloves and participants in the NSC group were asked to sit in front of the computer screen and wear the headphones for the sound. They were then briefed about the way they could interact with the application. Then, both groups viewed the application. The intervention lasted approximately 10 min for both groups. Participants completed the same post-intervention questionnaire immediately after viewing the application.

### Measures

Two questionnaires were given to the participants in total, which were the same for both groups, NSC and SC.

#### Pre-questionnaire

The pre-questionnaire was given to the participant before their exposure to the virtual world. Using this questionnaire, we gathered demographic data such as age, gender, occupation, hours spent playing video games, experience in using VR environments and computer programming knowledge.

It also included the following two measures:

##### Interpersonal reactivity index (IRI)

Interpersonal reactivity index is a self-report tool that consists of four 7-item subscales answered on a 5-point Likert scale ranging from “Does not describe me well” to “Describes me very well” (Davis, 1983), used to measure individual differences in empathy. The four subscales are: (a) Perspective-Taking scale (PT): the ability to shift perspectives when dealing with other people. (b) Empathic Concern scale (EC): assessment of the degree to which the respondent experiences feelings of warmth, compassion and concern for the observed individual. (c) Personal Distress scale (PD): measures the individual’s own feelings of fear, apprehension and discomfort at witnessing the negative experiences of others.

It is important to note, that in the pre-questionnaire, the fantasy scale was not measured, as the participants would experience through an application the life of the drug user, not imagine it.

##### Attitudes toward drug users

This was a 6-item attitudes scale on a 5-point Likert scale (1 = Completely disagree, 5 = Completely agree). It was adapted from the questionnaire used by Batson et al. (1997) and Herrera et al. (2018) to address attitudes toward drug users. Sample questions include “Our society should do more to protect the welfare of drug users” and “For more drug users, it is their fault that they are drug users.” Two out of the six questions were reverse coded. Higher scores indicate more positive attitudes toward drug users. This questionnaire was given to the participants before and after their exposure, in order to see if their exposure to the application, can change their attitudes toward drug users.

#### Post-questionnaire

The questionnaire given to all participants, regardless of their group, after their exposure included questionnaires measuring their level of empathy and personal distress, the Inclusion of the Other in the Self scale, the same questions regarding their attitudes toward drug users as the pre-questionnaire. It also included questions regarding presence [Place Illusion (PI) and Plausibility of the situation (Psi)], Body Ownership and Agency (BOA).

##### Empathy

Participants were asked to rate the extent to which they felt soft hearted, touched, sympathetic, or compassionate throughout the intervention by using a 7-point Likert scale (1 = Not at All, 7 = Very much). The results of these four questions were used to create an index of empathic concern. This measure was adapted from Batson et al. (1997).

##### Personal distress

Participants were asked to rate the extent to which they felt uneasy, troubled, distressed, or disturbed throughout the intervention by using a 7-point Likert scale (1 = Not at All, 7 = Very much). The results of these four questions were used to create an index of personal distress. This measure was also adapted from Batson et al. (1997).

##### IOS

The Inclusion of Other in the Self (IOS) scale measured how close the participants felt to the drug user. Developed by Aron et al. (1992), this scale depicts seven drawings of increasingly overlapping circles, anchored by the first picture of two non-overlapping circles and the seventh picture of two almost completely overlapping circles. Participants had to choose the picture that best represented the extent to which he/she felt connected to the drug user. The pictures are coded from 1 to 7 with the larger numbers indicating a closer relationship with the drug user.

##### Place illusion (PI) and plausibility of the situation and the virtual people

Place illusion (PI) refers to the sense of “being there” in a virtual world (Slater, 2009). PI was measured using a 5-item questionnaire on a 5-point Likert scale (1 = Not at all, 5 = Very much). Sample questions include “I had a sense of “being there” in the virtual environments” and “During the time of the experience, which was strongest overall, your sense of being in virtual environments, or of being in the real world of the laboratory?”

The Plausibility of the situation (Psi) is the illusion that what is apparently happening is really happening (even though you know that it is not). In the questionnaires, Psi was divided into two aspects; the Psi, and plausibility of the virtual human characters, which is if the participants felt that the virtual people were behaving, moving, reacting as if they were real. They were measured using a 6-itemand 5-item questionnaires, respectively, on a 5-point Likert scale (1 = Not at all, 5 = Very much). Sample questions for the plausibility of situation include “How much did you behave within the scenes as if the situations were real?” and for the plausibility of the virtual people “How much were you thinking things like “I know these people are not real” but then surprisingly finding yourself behaving as if they were?”

##### Body ownership and agency

According to Tsakiris et al. (2006) body ownership refers to the sense that one’s own body is the source of sensations. Gallagher (2000) defined the sense of agency as “the sense that I am the one who is causing or generating an action.” They were measured using a 4-item questionnaire on a 5-point Likert scale (1 = Completely agree, 5 = Completely disagree). Sample questions include “During the experience I felt as though I had two bodies” and “During the experience I felt that the virtual body belonged to someone else.” The complete questionnaires of all measures can be found in Supplementary Table S2.

## Results

All results were obtained with using the SPSS Software v.24 by IBM. All datasets generated for this study are included in the Supplementary Data Sheet S1.

The means and standard deviations for the outcome variables; IRI, Inclusion of the Other in the Self (IOS), Empathy, Personal Distress, Pre-, and Post-Attitudes toward drug users by condition (NSC and SC) are summarized in Table 1.

**TABLE 1:** Means and standard deviations for all outcome variables for both groups.

Measures	Condition			
	NSC		SC	
	Mean	*SD*	Mean	*SD*
IRI	71.1	11.02	77.4	11.08
IOS	2.85	1.89	3.75	1.55
Empathetic concern	19.4	6.87	19.6	5.68
Personal distress	17.8	6.57	19.55	6.19
Pre-attitudes	20.3	3.78	21.6	3.4
Post-attitudes	21.45	3.83	22.7	3.48

*SD, Standard deviation.*

### IRI

An independent-samples *t*-test was conducted to compare the level of empathy of the participants before their exposure in the two groups. Although the mean for the SC group is higher than the NSC group, there was not a statistically significant difference in the IRI scores for the NSC group (*M* = 71.1, *SD* = 11.02) and SC group (*M* = 77.4, *SD* = 11.08) conditions; *t*(38) = −1.802, *p* = 0.079.

Separately in the subscales, higher scores were shown was all three subscales of IRI for the SC group, but there was also no significant difference in participants’ trail-levels levels of empathy on any of the three subscales [empathetic concern (EC), perspective-taking (PT), and personal distress (PD)], of the IRI scales between the two conditions. More specifically, regarding the EC scale, for the NSC group (*M* = 26.20, *SD* = 5.22) and SC group (*M* = 28.45, *SD* = 4.61) conditions; *t*(38) = −1.443, *p* = 0.157. Next for the PT scale, for the NSC group (*M* = 25.90, *SD* = 4.59) and SC group (*M* = 27.20, *SD* = 3.95) conditions; *t*(38) = −0.959, *p* = 0.344. Finally for the PD scale, for the NSC group (*M* = 19.00, *SD* = 4.06) and SC group (*M* = 21.75, *SD* = 5.66) conditions; *t*(38) = −1.764, *p* = 0.086.

This suggests that the two conditions were balanced in terms of pre-intervention empathy and they were successfully randomly assigned into these two groups.

Gender differences were found in the SC group regarding IRI. More specifically, there was a significant difference between males (*M* = 71.11, *SD* = 11.12) and females (*M* = 82.54, *SD* = 8.34); *t*(18) = −2.627, *p* = 0.017. This suggests that females in the SC group had significantly higher levels of empathy than the males in the SC group before their exposure. More separately in the sub-scales, females only in the SC group had significantly higher levels of EC (*M* = 30.45, *SD* = 3.47) over the males (*M* = 26.00, *SD* = 4.82); *t*(18) = −2.401, *p* = 0.027 as well as levels of PD (*M* = 24.18, *SD* = 5.23) over the males (*M* = 18.77, *SD* = 4.89); *t*(18) = −2.365, *p* = 0.029. Females also had higher but not significantly different levels of PT (*M* = 27.90, *SD* = 3.70) over the males (*M* = 26.33, *SD* = 4.30); *t*(18) = −0.881, *p* = 0.390. This aligns with the findings of Davis (1980), where women displayed higher scores than men for each of the four subscales or IRI.

A Pearson product-moment correlation coefficient was computed to estimate the relationship between the IRI total score of the participants that was measured before their exposure and their attitudes toward drug users before and after their exposure. Results indicate significant positive correlation between these variables. More specifically, participants that reported more empathetic before their exposure also had more positive attitudes toward drug users after their exposure (positive medium correlation; *r* = 0.353, *n* = 40, *p* = 0.025) and before their exposure (positive strong correlation; *r* = 0.412, *n* = 40, *p* = 0.008). Separately in the two groups, this correlation was observed to a significant level only in the NSC group for their post-attitudes scores (*r* = 0.452, *n* = 20, *p* = 0.045) where a significant medium positive correlation was found.

### Attitudes Toward Drug Users

Both groups showed a significant difference in their reported attitudes toward drug users before and after their exposure. There was a significant difference in the attitudes of the participants in the NSC group for their attitudes before their exposure (*M* = 20.3, *SD* = 3.79) and after (*M* = 21.45, *SD* = 3.83); *t*(19) = −2.529, *p* = 0.020. Similarly, there was a significant difference in the attitudes of the participants in the SC group for their attitudes before their exposure (*M* = 21.6, *SD* = 3.4) and after (*M* = 22.7, *SD* = 3.48); *t*(19) = −2.125, *p* = 0.047.

Gender differences were found in the NSC group regarding both pre-attitudes and post-attitudes. More specifically, there was a significant difference regarding the pre-attitudes of males (*M* = 18.75, *SD* = 3.57) and females (*M* = 22.62, *SD* = 2.92); *t*(18) = −2.546, *p* = 0.020. Also, there was a significant difference regarding the post-attitudes too, of males (*M* = 20.08, *SD* = 3.89) and females (*M* = 23.50, *SD* = 2.82); *t*(18) = −2.127, *p* = 0.047. Both these results suggest that females in the NSC group had more positive attitudes toward drug users both before and after their exposure than the males in the NSC group.

There was not a significant difference between the two groups in their post-attitudes, NSC (*M* = 21.45, *SD* = 3.83) and SC (*M* = 22.7, *SD* = 3.48) conditions; *t*(38) = −1.08, *p* = 0.287.

### IOS

**FIGURE 4:**
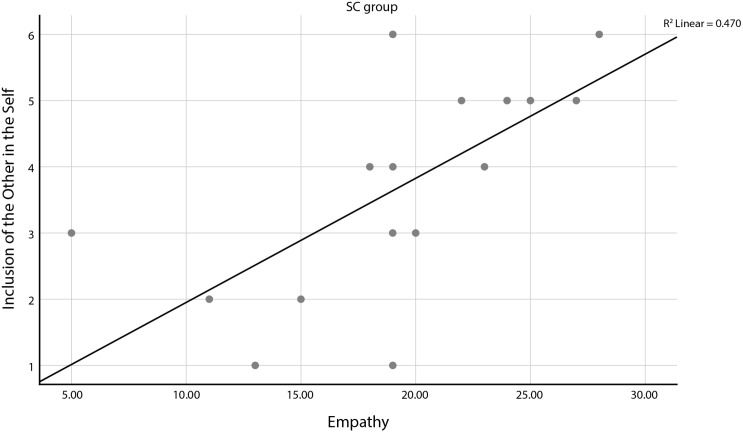
Strong correlation between closeness to the drug user and empathy in the SC group.

There was not a significant difference in the closeness of the participants toward the drug user between the two groups, NSC (*M* = 2.85, *SD* = 1.899) and SC (*M* = 3.75, *SD* = 1.552) conditions; *t*(38) = 0.084, *p* = 0.109. Regarding the SC group, IOS was positively significantly correlated to their reported levels of post-attitudes (*r* = 0.512, *n* = 20, *p* = 0.021) and their reported levels of empathy (Figure 4) (*r* = 0.686, *n* = 20, *p* = 0.001). Which means that participants in the SC group who reported being emotionally closer to the drug user, also reported more positive attitudes toward drug users after their exposure, and more empathetic.

Regarding the NSC group, IOS was positively significantly correlated only to their reported levels of BoA (*r* = 0.508, *n* = 20, *p* = 0.022). Which means that participants in the NSC group who reported being emotionally closer to the drug user, also reported higher levels of BOA.

### Empathy

Participants empathy was measured after their exposure. The overall reliability of the index was good, Cronbach’s alpha = 0.921. There was not a significant difference in the empathy levels between the two groups, NSC (*M* = 19.4, *SD* = 6.87) and SC (*M* = 19.6, *SD* = 5.68) conditions; t (38) = −0.100, *p* = 0.921.

Regarding the NSC group; empathy was positively significantly correlated to their reported levels of BoA (*r* = 0.475, *n* = 20, *p* = 0.034), their reported levels of PVP (*r* = 0.564, *n* = 20, *p* = 0.010) and finally their personal distress levels (*r* = 0.630, *n* = 20, *p* = 0.003). This means that the more empathetic they reported they felt after their exposure, the more distressed they were and reported higher levels of body ownership, agency and plausibility of the virtual people.

### Personal Distress

Participants personal distress was measured after their exposure. The overall reliability of the index was good, Cronbach’s alpha = 0.889. There was not a significant difference in the personal distress levels between the two groups, NSC (*M* = 17.8 *SD* = 6.57) and SC (*M* = 19.55, *SD* = 6.19) conditions; *t*(38) = −0.866, *p* = 0.392.

Regarding the NSC group, personal distress was positively significantly correlated to their reported levels of PI (*r* = 0.457, *n* = 20, *p* = 0.043), their reported levels of PSI (*r* = 0.514, *n* = 20, *p* = 0.020) and their levels of PVP (*r* = 0.513, *n* = 20, *p* = 0.021). This means that the more distressed they felt, the higher the levels of PI, Psi, and the virtual people.

### Factor Analysis

For the questionnaire data regarding the participants experience, factor analysis was carried out to reduce the number of questionnaire variables, which also has the advantage of transforming ordinal variables to continuous ones. Corresponding factor scores were used, and the interpretation of each factor was identified. Subsequent analysis on the derived variables was done using independent samples *t*-test.

**FIGURE 5:**
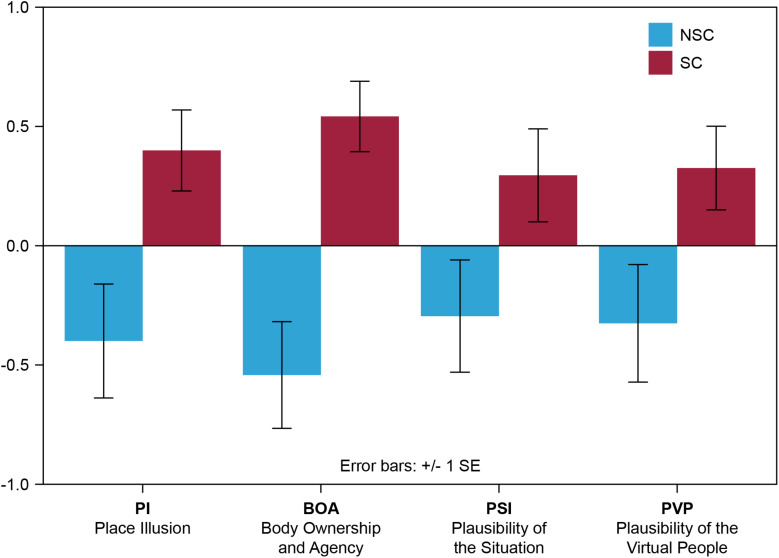
Means and standard errors of the derived factor scores for all four variables PI, BOA, PSI, and PVP.

Figure 5 shows the bar chart of the means and standard errors of the derived factor scores for all four variables Place Illusion (PI), Body Ownership and Agency (BOA), Plausibility of the situation (Psi), and Plausibility of the virtual people (PVP).

#### Place Illusion (PI)

Factor analysis in the questionnaire on the participants reported PI has resulted in a single factor. The factor loadings of the scoring variable PI are shown in Table 2. The factor is interpreted as “the illusion of being there.” There was a statistically significant difference between the two groups. Participants in the SC condition reported higher levels of presence (0.399 ± 0.169) compared to participants in the NSC condition (−0.399 ± 0.238). An independent samples *t*-test showed that the above differences are significant [*t*(38) = −2.728, *p* = 0.010].

**TABLE 2:** Factor analysis for Place Illusion, resulting in a single factor F1 and the corresponding scoring coefficients of the factor score yp1.

Variable	Factor loadings	Scoring coefficients
	F1	yp1
There	0.886	0.257
Real	0.808	0.234
Visited	0.787	0.228
Lab	0.815	0.236
Overwhelm	0.854	0.247

#### Body Ownership and Agency (BOA)

Factor analysis in the questionnaire on the participants reported BOA has resulted in a single factor. The factor loadings of the scoring variable BOA are shown in Table 3. The factor is interpreted as “the sense that one’s own body is the source of sensations and the sense that I am the one who is causing or generating an action.” There was a statistically significant difference between the two groups. Participants in the SC condition reported higher levels of BOA (0.541 ± 0.147) compared to participants in the NSC condition (−0.541 ± 0.223). An independent samples *t*-test showed that the above differences are significant [*t*(38) = −4.047, *p* = 0.000].

**TABLE 3:** Factor analysis for Body Ownership and Agency, resulting in a single factor F1 and the corresponding scoring coefficients of the factor score yboa1.

Variable	Factor loadings	Scoring coefficients
	F1	yboa1
Mybody	0.924	0.393
Twobodies	0.426	0.181
Agency	0.892	0.379
Otherbody	−0.721	−0.307

#### Plausibility of the Situation (Psi)

Factor analysis in the questionnaire on the participants reported Psi has resulted in a single factor. The factor loadings of the scoring variable PSI are shown in Table 4. The factor is interpreted as “the illusion that events are actually happening.” Participants in the SC condition rated their sensed illusion more positively (0.294 ± 0.194) compared to participants in the NSC condition (−0.294 ± 0.235). An independent samples *t*-test showed that the above differences are not significant [*t*(38) = −1.930, *p* = 0.061].

**TABLE 4:** Factor analysis for Plausibility of the situation, resulting in a single factor F1 and the corresponding scoring coefficients of the factor score ypsi1.

Variable	Factor loadings	Scoring coefficients
	F1	ypsi1
Behavereal	0.911	0.196
Emotionreal	0.881	0.213
Thoughtsreal	0.823	0.199
Behaveasifreal	0.821	0.199
Physicalreal	0.807	0.196
Experiencereal	0.833	0.202

#### Plausibility of the Virtual People (PVP)

Factor analysis in the questionnaire on the participants reported PVP has resulted in a single factor. The factor loadings of the scoring variable PVP are shown in Table 5. The factor is interpreted as the “behavioral, physiological, emotional, and thinking responses as if the people were real.” There was a statistically significant difference between the two groups. Participants in the SC condition reported higher levels of PVP (0.541 ± 0.147) compared to participants in the NSC condition (−0.541 ± 0.223). An independent samples *t*-test showed that the above differences are significant [*t*(38) = −4.047, *p* = 0.000].

**TABLE 5:** Factor analysis for Plausibility of the virtual people, resulting in a single factor F1 and the corresponding scoring coefficients of the factor score ypvp1.

Variable	Factor loadings	Scoring coefficients
	F1	ypvp1
Mybody	0.924	0.393
Twobodies	0.426	0.181
Agency	0.892	0.379
Otherbody	−0.721	−0.307

## Discussion

The aim of this study was to investigate the role of supported sensorimotor contingencies when attempting to promote empathy and positive attitudes toward drug users, by comparing a VR system that offered perception through sensorimotor contingencies (SC condition) and system that didn’t offer that (NSC condition), by showing the participants scenes from the life of a drug user.

Although the SC group had higher means in all the measures, as seen in Table 1, they were not significantly different to those of the NSC group. This aligns with the results of Herrera et al. (2018) that showed that the differences between mediated perspective-taking tasks, regardless of how immersive they are, were not significant in terms of IOS, empathy or personal distress. Both groups showed a significant difference in their reported attitudes toward drug users before and after their exposure which means that both interventions succeeded in eliciting more positive attitudes toward drug users to the participants. Participants in the VR group reported significantly higher levels of PI, BOA, and PVP but not the situation. The results could be explained by the fact that most of the participants (60%) had non or little experience with VR and maybe this new experience and technology distracted them from experiencing fully the scenario, resulting in similar levels of empathy and personal distress. The SC groups’ higher levels of empathy and body ownership could explain the correlation between their closeness to the drug user and empathy as well as their positive attitudes toward drug users. This is in line with the study by Maister et al. (2013) that showed the more intense the participants’ illusion of ownership over a dark-skinned rubber hand, the more positive their implicit racial attitudes. Additionally, the experience lasted approximately 6 min, and this perhaps was not enough time for the participants to truly experience what it is like to be a drug user.

Furthermore, this study aimed to show the participants, that in most cases, people end up doing drug use because of bad situations that are going through in life, and they find “relief” and an “escape” in drug use. Considering that, the scenes and scenario created, wanted to reflect the journey a person goes in, before drug use and after, and the consequences it has in his health, life, family and workspace. Future studies should focus more on the negative aspects that drug use has on drug users and their life. Future studies should also investigate the role of agency and interactivity plays in empathy and attitudes toward stigmatized people. Interactivity has been found to increase empathy (Vorderer et al., 2001), and it can be utilized in VR applications. As many companies are releasing VR headsets every year with way more features and needing less extra equipment to allow hands and even body tracking, it would be interesting to compare in future studies, the difference between low vs. high cost VR and tracking equipment in the supporting sensorimotor contingencies. Examples of that could include the comparison of motion tracking technologies like the more affordable Microsoft Kinect and a more expensive specialized motion tracking suit like the one used by the participants of the SC group in the current study, or even different cost HMD’s which offer less or more tracking possibilities.

The findings of this study provide more evidence that show that VRPT, can be used for to induce empathy and prosocial behavior of the participants toward members of stigmatized groups. Further research is needed to investigate how perception through sensorimotor contingencies can be exploited to the fullest to be used as an effective method to induce empathy and change attitudes toward stigmatized by society people.

## Data Availability Statement

All datasets generated for this study are included in the article/Supplementary Material.

## Ethics Statement

The study has been approved by the Research’s Ethics and Deontology Committee of the Cyprus University of Technology. The participants provided their written informed consent to participate in this study. Written informed consent was obtained from the individual(s), and minor(s)’ legal guardian/next of kin, for the publication of any potentially identifiable images or data included in this manuscript.

## Author Contributions

MC made substantial contribution in the conception and design of the study, development of the VR application, data collection, analysis and interpretation of the data, and drafting the article. DM-G contributed in the design and conception of the study, interpretation of the data, revising critically the manuscript, and supervised and coordinated all the steps of the study. CK contributed partially in the development of the VR application and substantially in data analysis.

## Conflict of Interest

The authors declare that the research was conducted in the absence of any commercial or financial relationships that could be construed as a potential conflict of interest.

## Supplementary Material

TABLE S1 Additional views from the various scenes.

TABLE S2 Participant’s demographic data and questionnaires.

DATA SHEET S1 All datasets generated for this study.

VIDEO S1 A video illustrating the application.
