# Supplementary material for: A Virtual Reality Simulation of Drug Users’ Everyday Life: The Effect of Supported Sensorimotor Contingencies on Empathy
Source: Front Psychol. 2020 Jun 5;11:1242. doi: 10.3389/fpsyg.2020.01242 (PMC7289998; doi:10.3389/fpsyg.2020.01242)

A Virtual Reality Simulation of Drug Users' Everyday Life: the Effect of Supported Sensorimotor Contingencies on Empathy

Maria Christofi, Despina Michael-Grigoriou, Christos Kyrlitsias

Supplementary Material - Table 1:
Additional views from the various scenes

**Figure S1.** Additional views from Scene 1
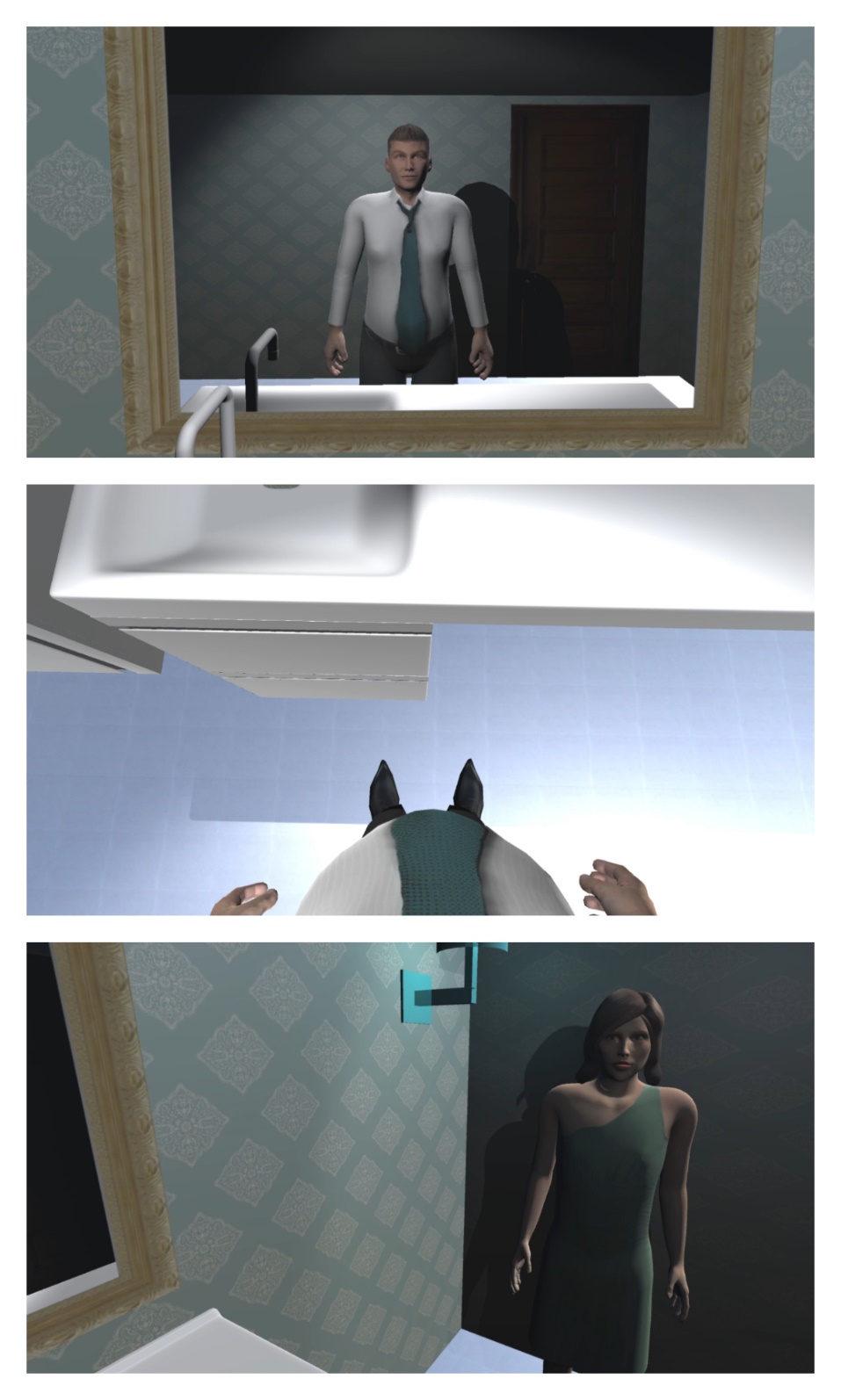


**Figure S2.** Additional views from Scene 2


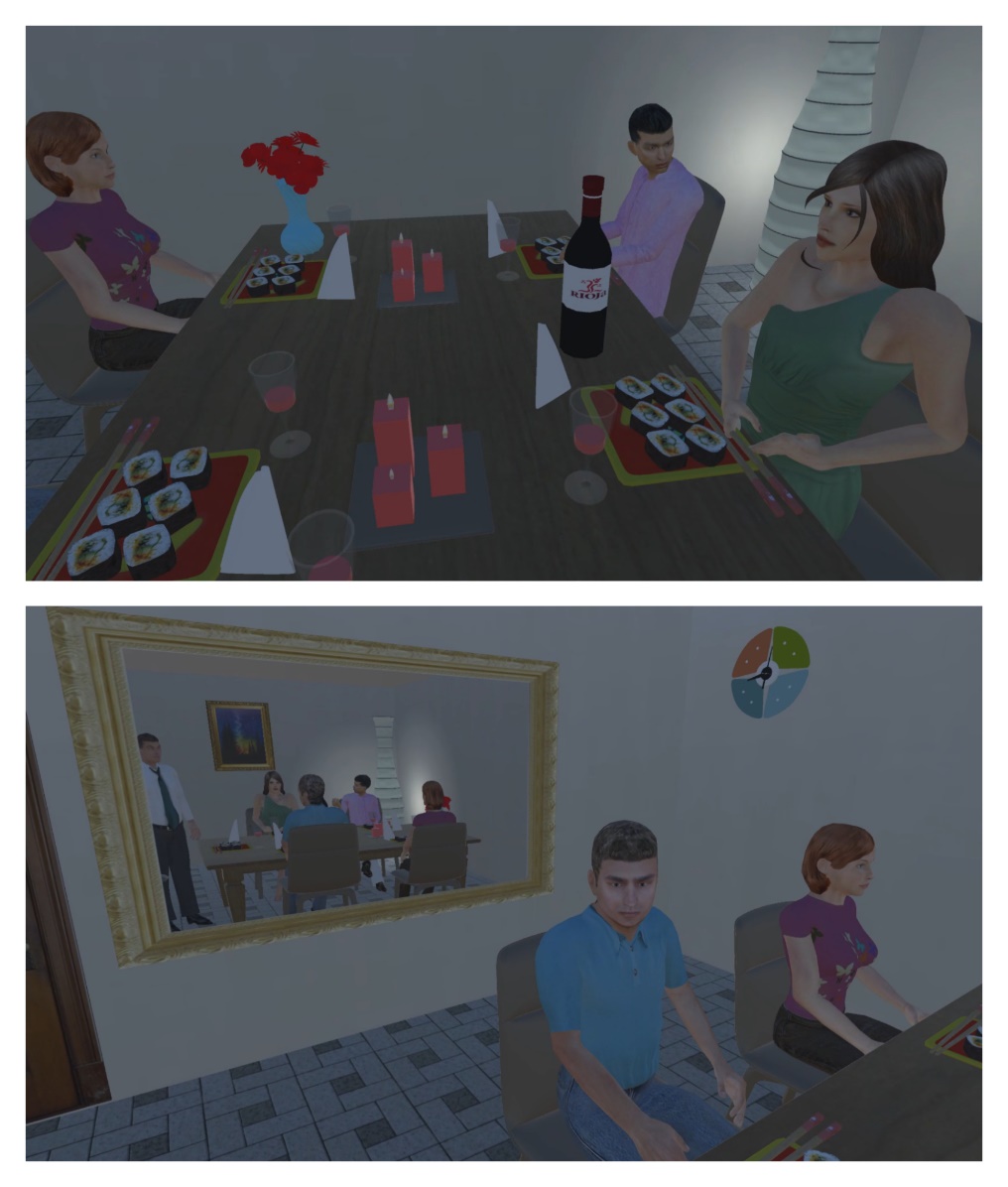


**Figure S3.** Additional views from Scene 3

##
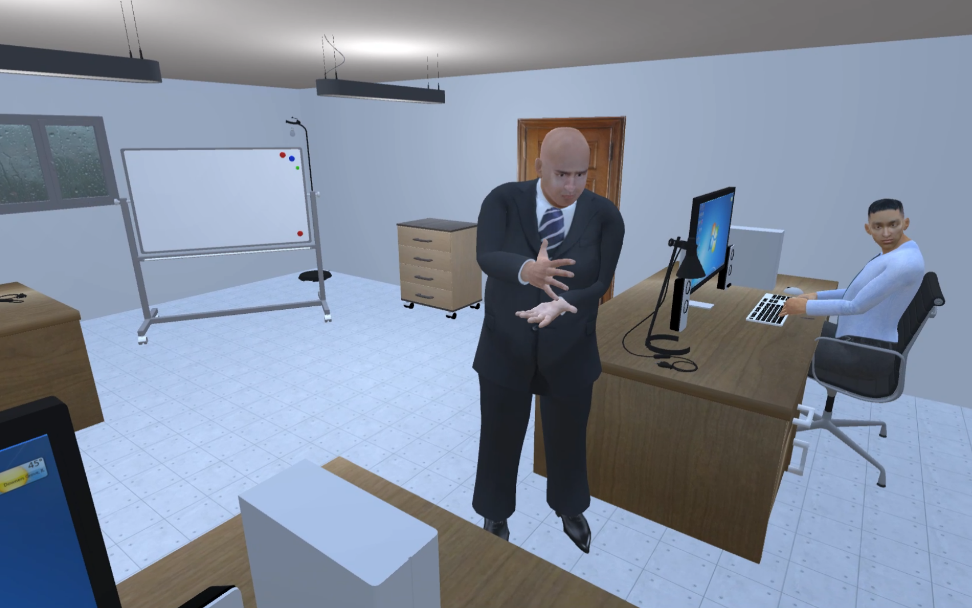


**Figure S4.** Additional views from Scene 4


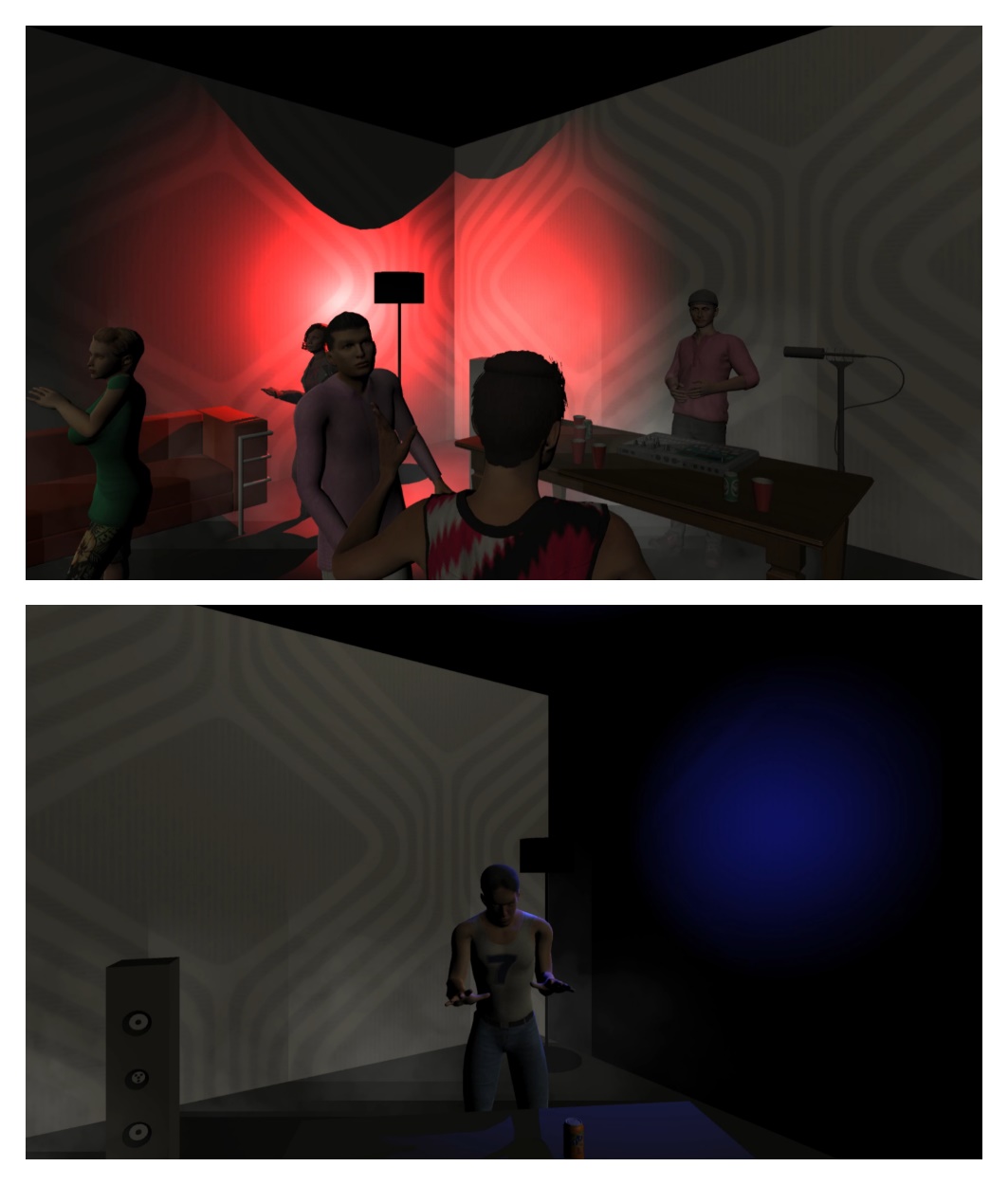


**Figure S5.** Additional views from Scene 5


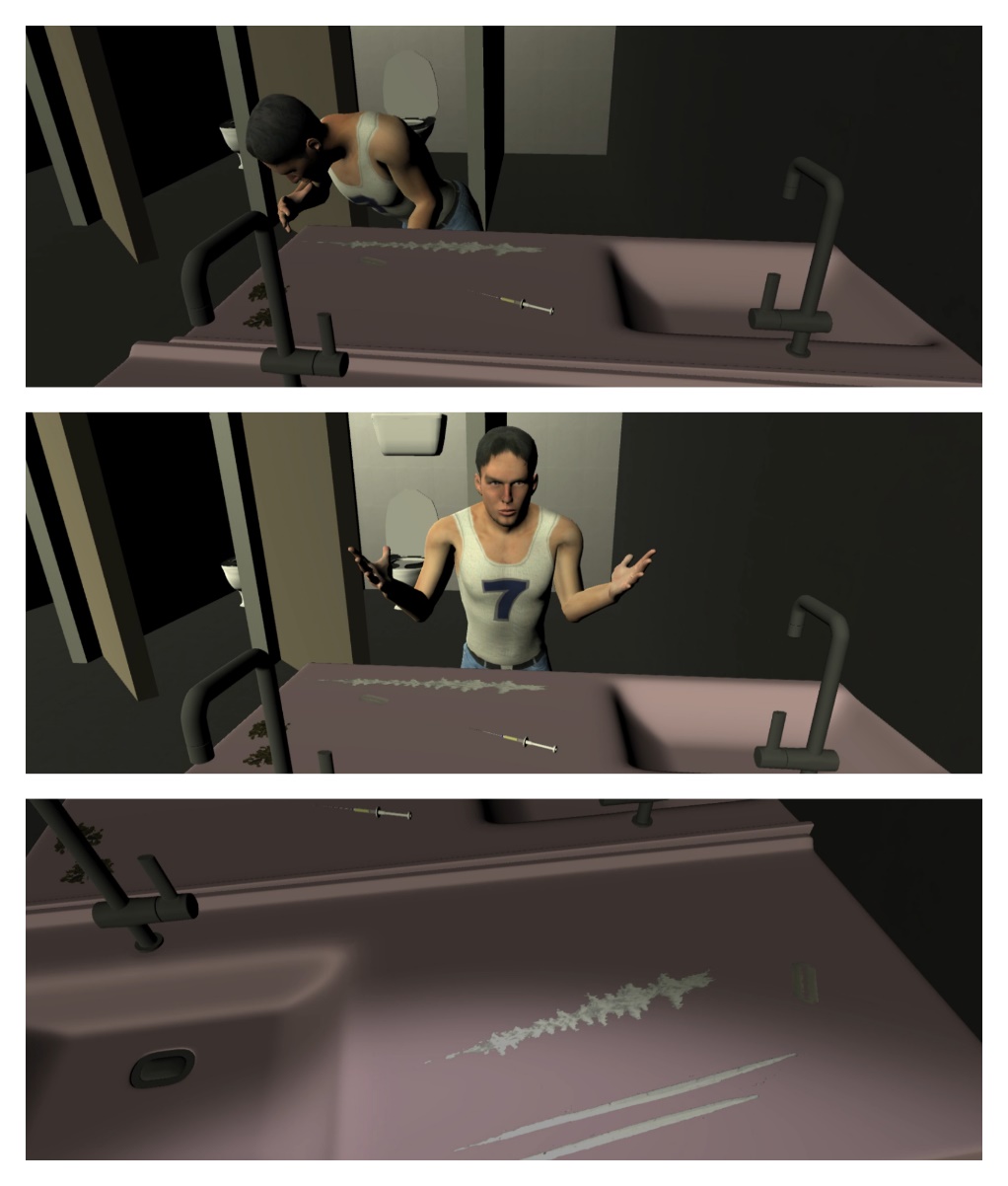


**Figure S6.** Additional view from Scene 6


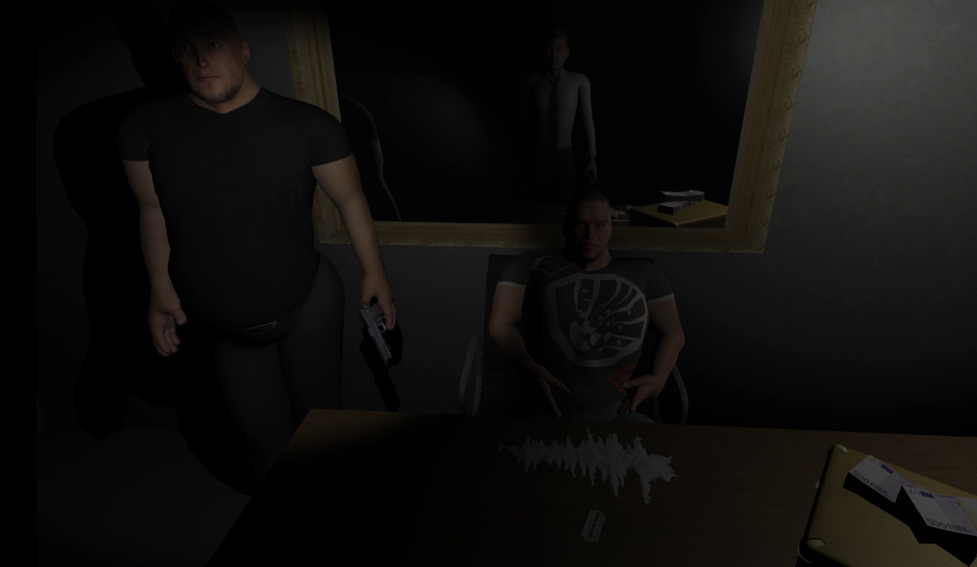


**Figure S7.** Additional view from Scene 7


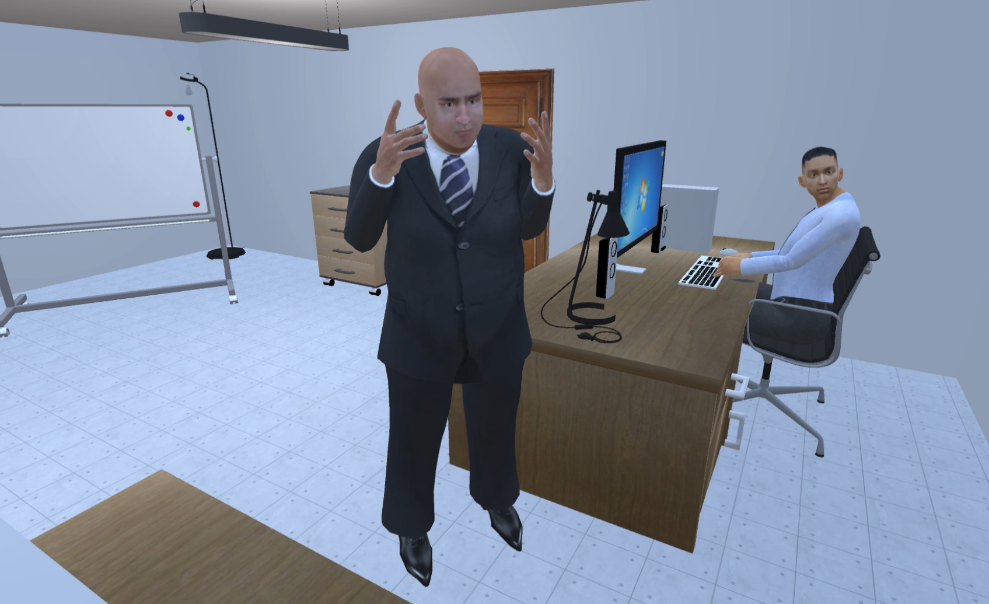


**Figure S8.** Additional views from Scene 8
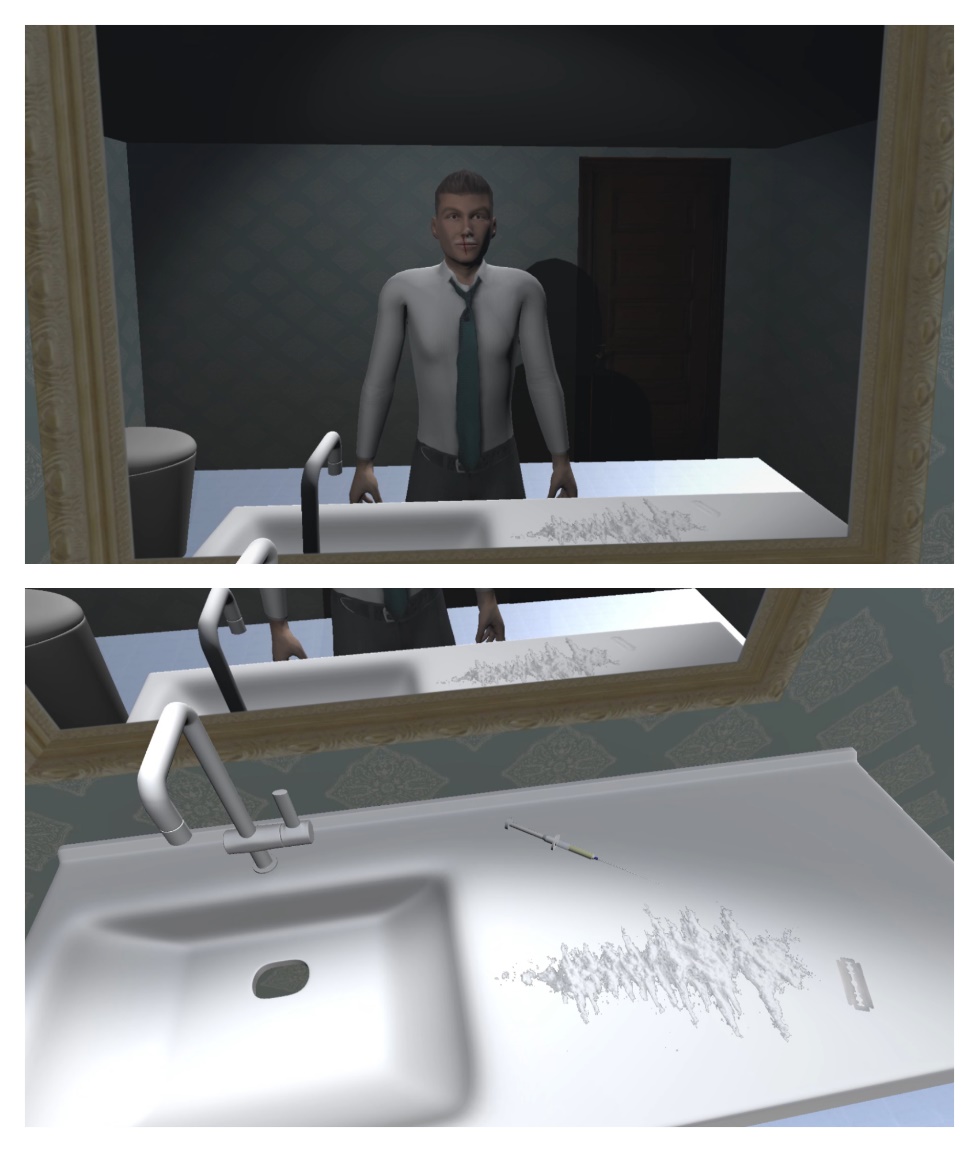


**Figure S9.** Additional views from Scene 9


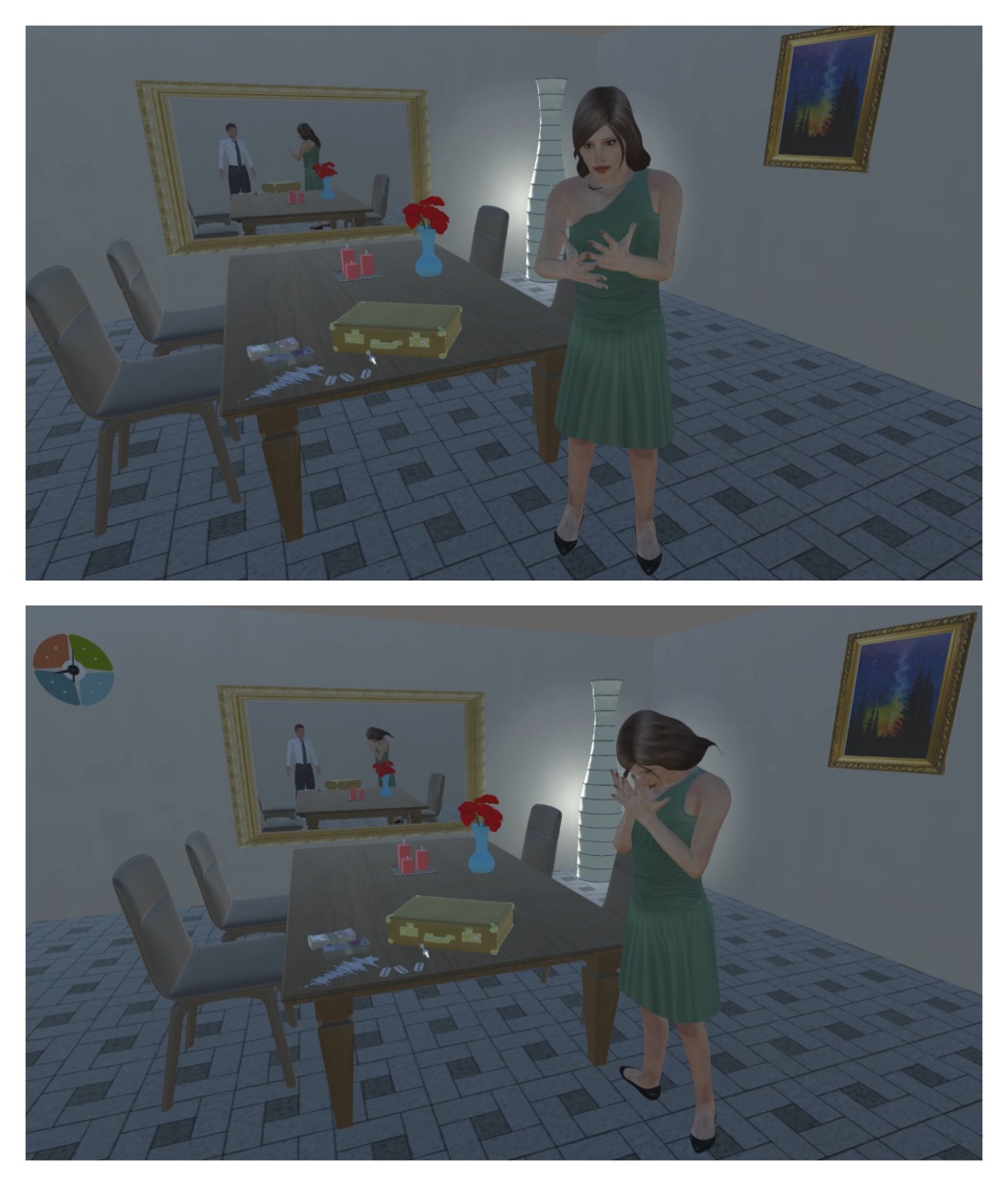

Supplement: TABLE S1 — Additional views from the various scenes. [file Table_1.docx]
