# Supplementary material for: A Virtual Reality Simulation of Drug Users’ Everyday Life: The Effect of Supported Sensorimotor Contingencies on Empathy
Source: Front Psychol. 2020 Jun 5;11:1242. doi: 10.3389/fpsyg.2020.01242 (PMC7289998; doi:10.3389/fpsyg.2020.01242)
Supplement: TABLE S2 — Participant’s demographic data and questionnaires. [file Table_2.docx]

A Virtual Reality Simulation of Drug Users' Everyday Life: the Effect of Supported Sensorimotor Contingencies on Empathy

Maria Christofi, Despina Michael-Grigoriou, Christos Kyrlitsias

Supplementary Material - Table 2:
Participants’ Demographic Data and Questionnaires

**Table S1.** Frequencies of demographics across the two groups.

| **Demographics** |  | **NSC Group** | **SC Group** |
| --- | --- | --- | --- |
| **Age** | 18-24 | 11 | 6 |
|  | 25-29 | 1 | 8 |
|  | 30-39 | 6 | 5 |
|  | 50-59 | 2 | 1 |
| **Gender** | Male | 12 | 9 |
|  | Female | 8 | 11 |
| **Occupation** | Student | 12 | 5 |
|  | Public Sector | 3 | 2 |
|  | Private Sector | 3 | 7 |
|  | Teacher/Professor | 2 | 5 |
|  | Other | 0 | 1 |
| **Hours spent playing video games, per week** | 0 hours | 5 | 5 |
|  | <1 hour | 3 | 8 |
|  | 1-3 hours | 7 | 1 |
|  | 3-5 hours | 1 | 1 |
|  | 5-7 hours | 0 | 3 |
|  | 7-9 hours | 0 | 0 |
|  | >9 hours | 4 | 2 |
| **Experience in using Virtual Reality environments** | Not at all | 4 | 3 |
|  | A little | 11 | 6 |
|  | Moderately | 2 | 1 |
|  | Much | 2 | 5 |
|  | Very much | 1 | 5 |
| **Computer programming knowledge** | Novice | 3 | 1 |
|  | Beginner | 11 | 5 |
|  | Competent | 3 | 8 |
|  | Proficient | 2 | 6 |
|  | Expert | 1 | 0 |

**Table S2.** The 5-item questionnaire for Place Illusion (PI)

| **Place Illusion** |
| --- |
| I had a sense of “being there” in the virtual environments: |
| There were times during the experience when the virtual environments were the reality for me… |
| When you think back about your experience, do you think of the virtual environments more as images that you saw, or more as somewhere that you visited? |
| During the time of the experience, which was strongest overall, your sense of being in virtual environments, or of being in the real world of the laboratory? |
| During the time of the experience, did you often think to yourself that you were just sitting in a laboratory or did the experience overwhelm you? |

**Table S3.** The 6-item questionnaire for the Plausibility of the Situation (PSI)

| **Plausibility of the Situation** |
| --- |
| How much did you behave within the scenes as if the situations were real? |
| How much was your emotional response the same as if it had been real? |
| How much were the thoughts you had within the experience the same as if it had been a real situation? |
| How much were you thinking things like “I know this isn’t real” but then surprisingly finding yourself behaving as if it was real? |
| To what extent were your physical responses within the experience (e.g., heart rate, blushing, sweating, etc.) the same as if it had been a real situation? |
| Overall, how much did you treat the experience as if it were real? |

**Table S4.** The 5-item questionnaire for the Plausibility of the Virtual People (PVP)

| **Plausibility of the Virtual People** |
| --- |
| How much did you behave as if the virtual people were real? |
| How much was your emotional response to the virtual people as if they were real? |
| How much were your thoughts in relation to the virtual people as if they were real? |
| How much did you have physical responses (such as change in heart rate, blushing, sweating, etc.) to the virtual people as if they were real? |
| How much were you thinking things like “I know these people are not real” but then surprisingly finding yourself behaving as if they were? |

**Table S5.** The 4-item questionnaire for the Body Ownership and Agency (BOA)

| **Body Ownership and Agency** |
| --- |
| During the experience I felt that the body I saw when looking down toward myself was my own body (even though it didn’t look like me) |
| During the experience I felt as though I had two bodies |
| During the experience I felt that the movements of the virtual body were my movements |
| During the experience I felt that the virtual body belonged to someone else |
